# Supplementary material for: Three-Dimensional Cephalometric Landmarking and Frankfort Horizontal Plane Construction: Reproducibility of Conventional and Novel Landmarks
Source: J Clin Med. 2021 Nov 15;10(22):5303. doi: 10.3390/jcm10225303 (PMC8624462; doi:10.3390/jcm10225303)
Supplement: Supplementary file 1 [file jcm-10-05303-s001.zip › jcm-1404520-supplementary.pdf]

Three-Dimensional Cephalometric Landmarking and Frankfort Horizontal Plane Construction: Reproducibility of Conventional and Novel Landmarks.

Dot, G.; Rafflenbeul, F.; Kerbrat, A.; Rouch, P.; Gajny, L.; Schouman, T.

J. Clin. Med. 2021

Supplementary Material 1

Written instructions for landmarking process
Mimics software

Useful Mimics commands:

3D view	Rotation of the object	Mouse right click + move near the object
	Tilt of the object	Mouse right click + move far from the object
	Translation of the object	Shift + right mouse click + move
	Zoom in/out	Ctrl + right mouse click + move<br>or Mouse scroll
2D views	Translation of the slice	Shift + right mouse click + move
	Zoom in/out	Ctrl + right mouse click + move
	Change slice	Mouse scroll
	Change contrast	Alt + right mouse click + move

Procedure

- Open Mimics document
- If needed, change document’s layout to get a bigger 3D view: View > Layouts > Horizontal
- On the right panel, hide all masks (eye icon) & all objects other than Upper Skull / Mandibula / Upper Teeth / Lower Teeth
- Orient the skull object for it to be approximately aligned with Frankfort plane
- Launch Analyze > Measure and Analyze and choose “Full ceph analysis”

- Start Time measurement (chronometer)

- Follow the order of the landmarks to annotate (1:, 2:, 3:.... to 33:)
- If a landmark is missing (e.g. missing tooth), do not annotate it

1: Nasion	Medial (and upper) point of the fronto-nasal suture	3D view, frontal<br>Check on 2D slices if necessary (axial/sagittal)
2,3: Orbitale L/R	Lowest point of the orbitale rim L/R	3D view, frontal and check from above that the points are well on the orbital rim

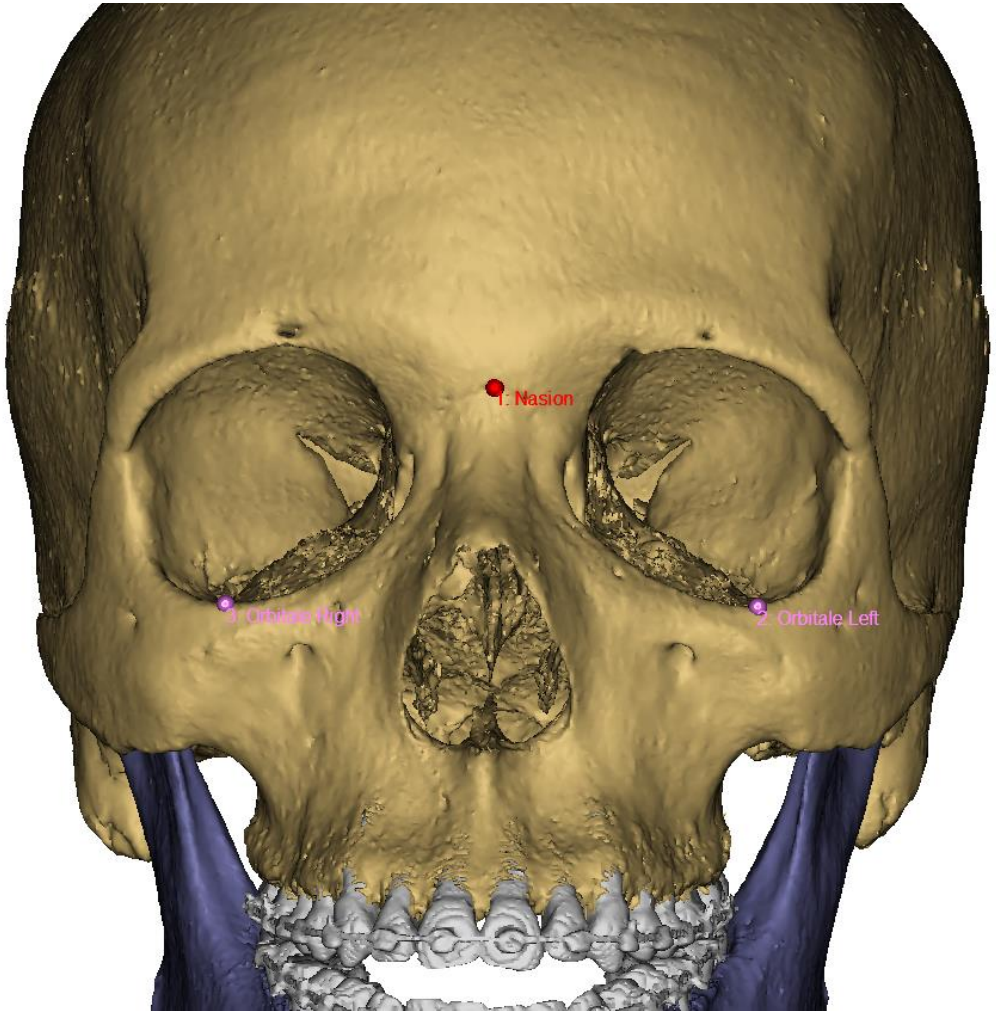

4,5: Infraorbital Foramen L/R	External & most distal point of the infraorbital foramen L/R	3D view, ¾ mesio-lateral view<br>Check on 2D slice (Axial)

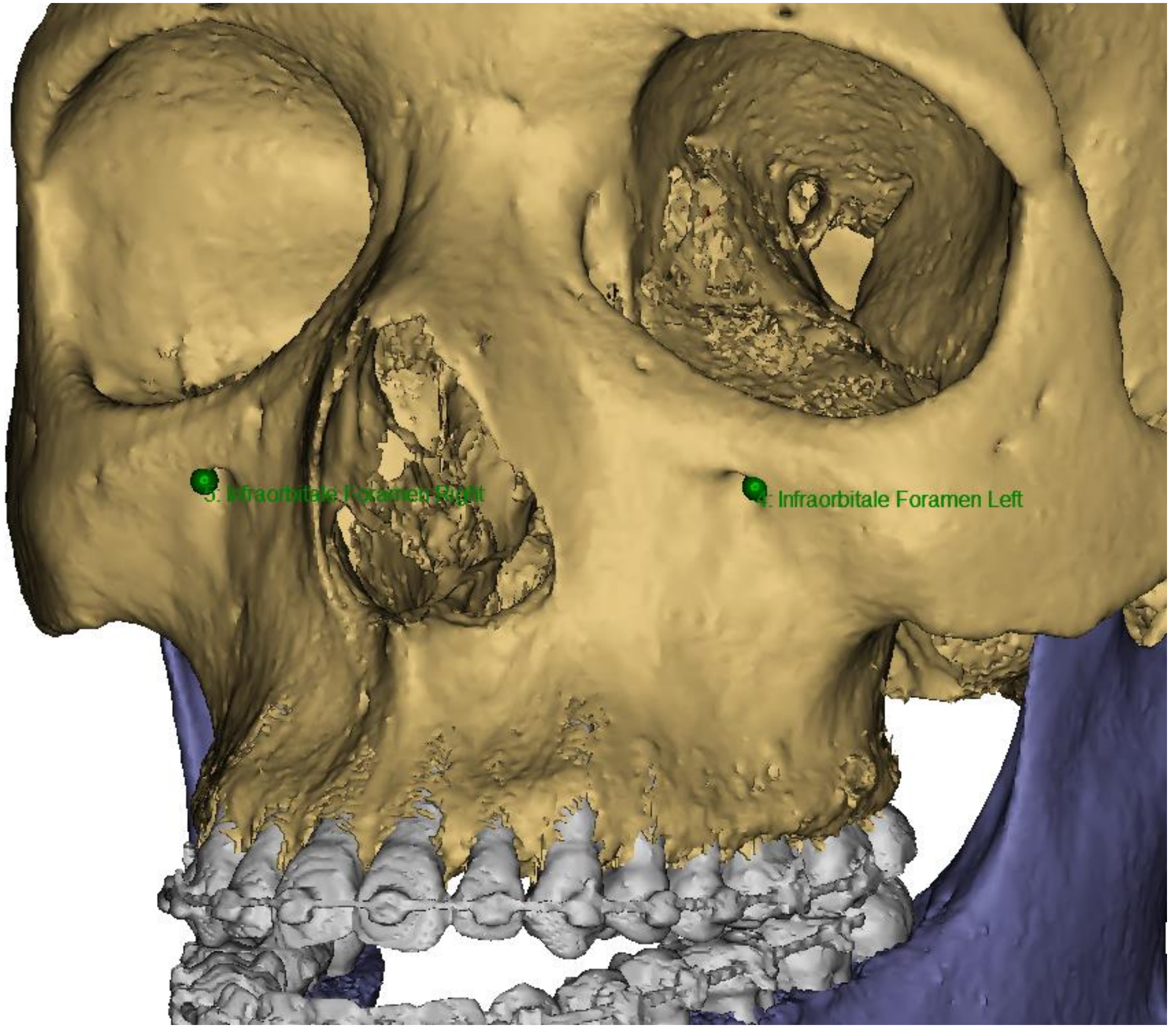

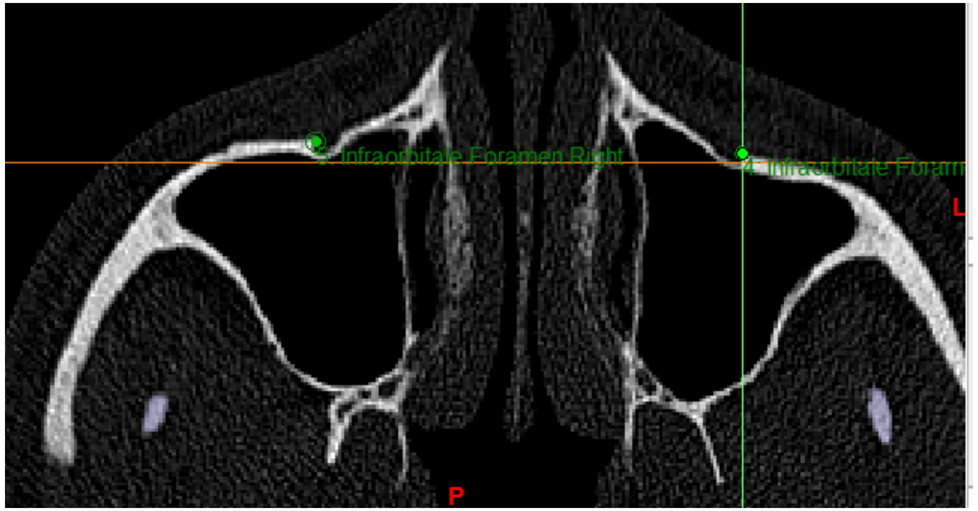

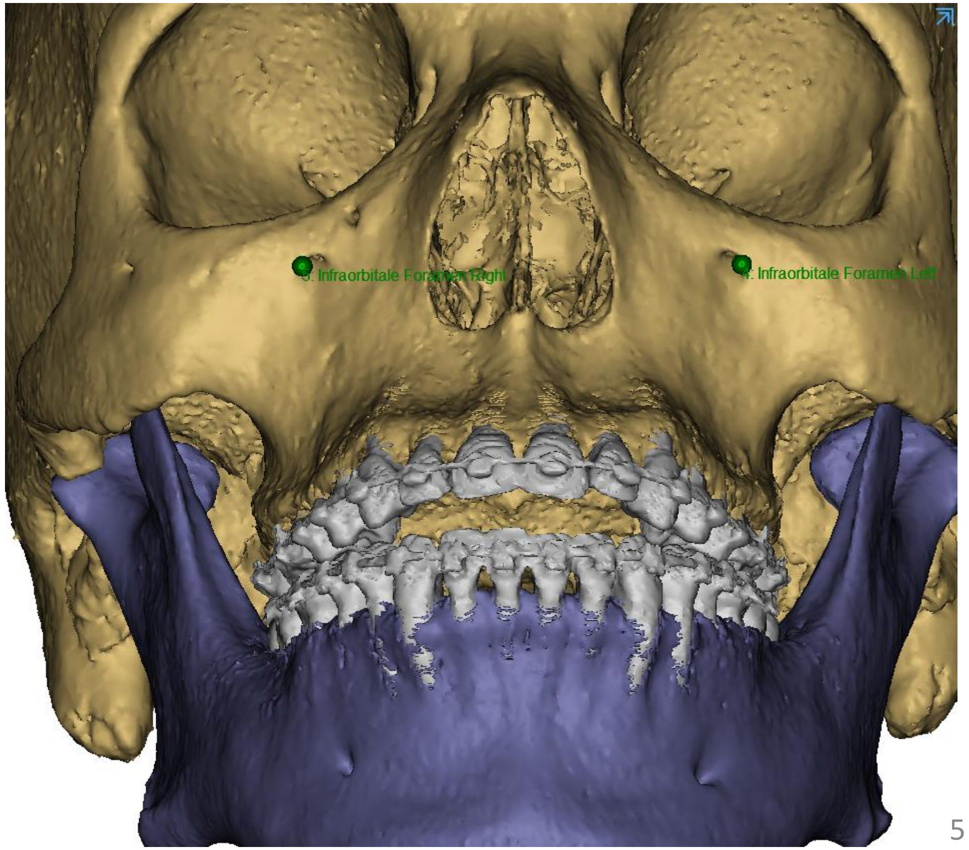

6: ANS	Medial and most anterior point of the nasal spine	3D view, frontal<br>Check on 2D slices (axial/sagittal)<br>In case of bifid spine, stay at a medial position
7: A	Medial & most posterior point of the maxilla	3D view, frontal<br>Check afterwards laterally (see after point 18)
8: B	Medial & most osterior point of the mandible	3D view, frontal<br>Check afterwards laterally (see after point 18)
9: Pog	Medial and most anterior point of the mandible	3D view, frontal<br>Check afterwards laterally (see after point 18)
10: Gnathion	Medial & mid-point between Pog and Me	3D view, frontal<br>Check afterwards laterally (see after point 18)
11: Menton	Medial and lowest point of the mandible	3D view, inferior<br>Check afterwards laterally (see after point 18)

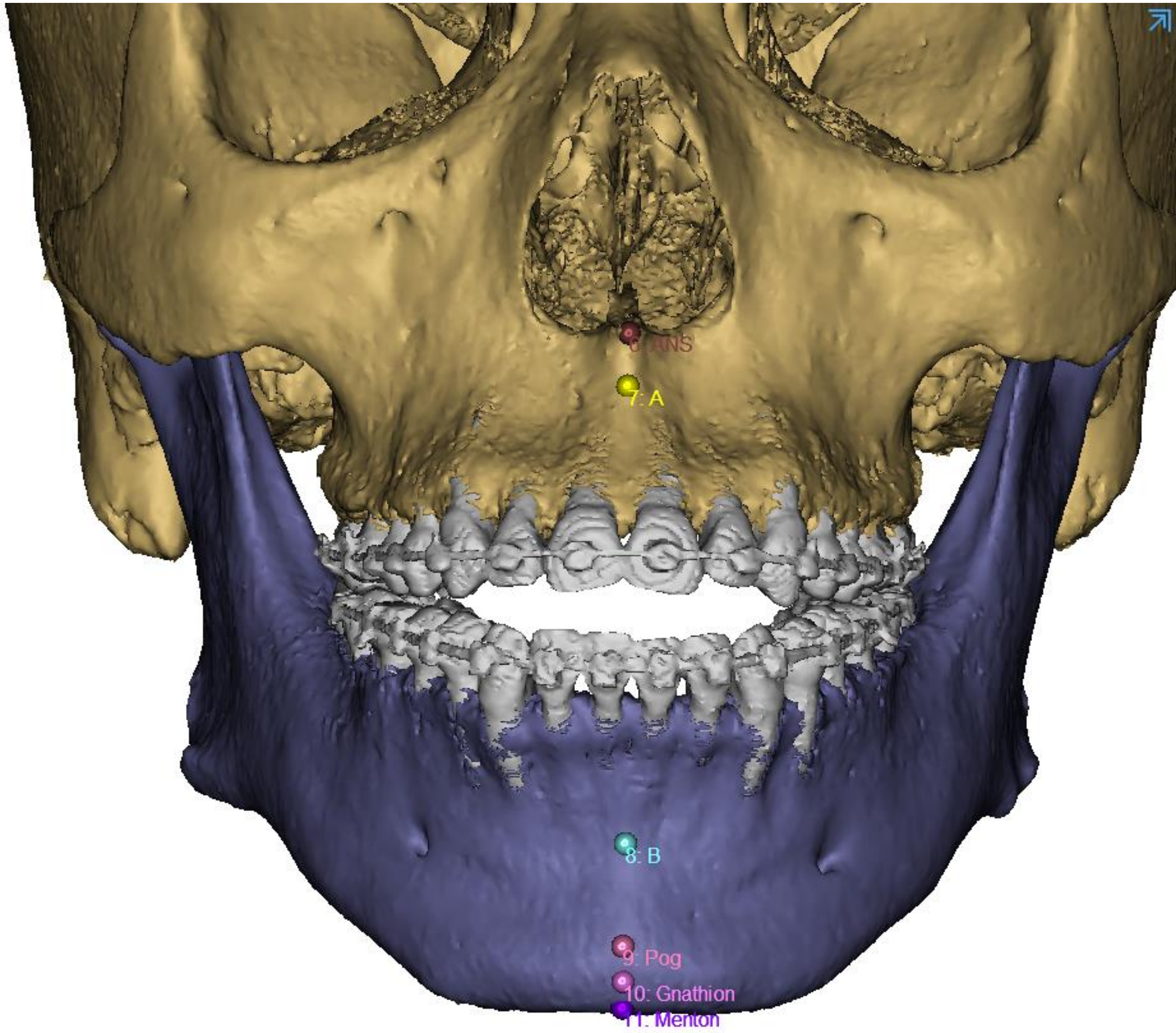

12, 13: Mental Foramen L/R	External most mesial point of the mental foramen L/R	3D view, ¾ disto-lateral view<br>Check on 2D slice (Axial)

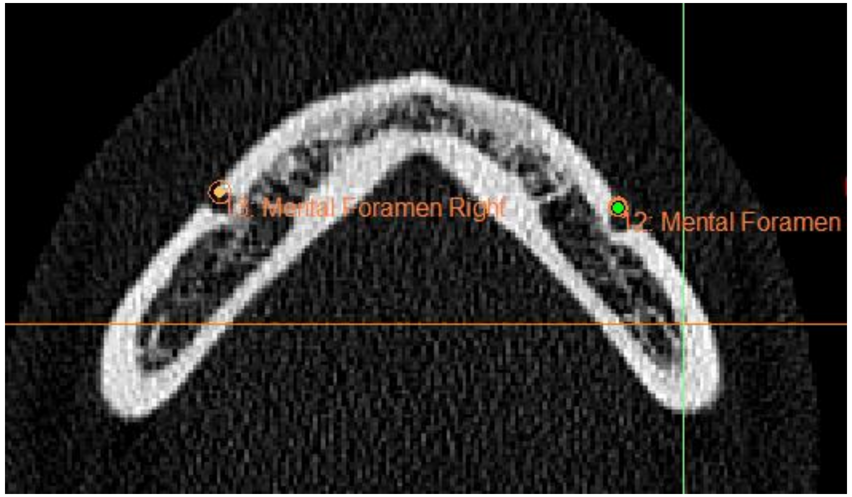

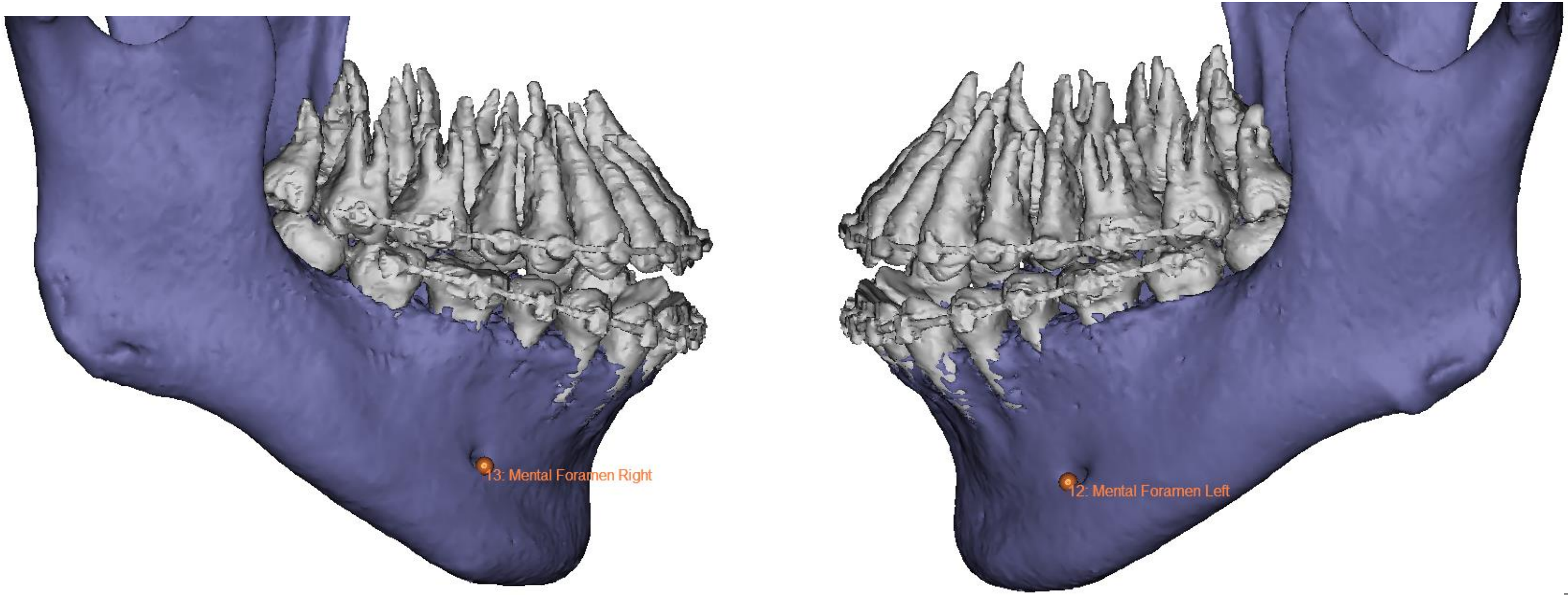

14,15: Gonion L/R	Mid-point of the gonial angle L/R	3D view, lateral. Mid-point between the beginning and the end of the mandibular angle<br>Check from behind that the points are well located on the mandibular external border<br>Before placing R one it can be easier to hide L one

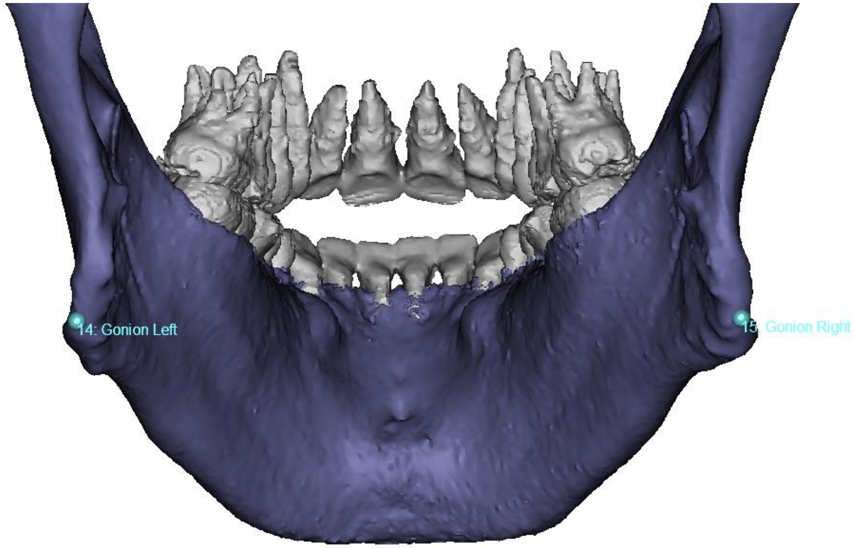

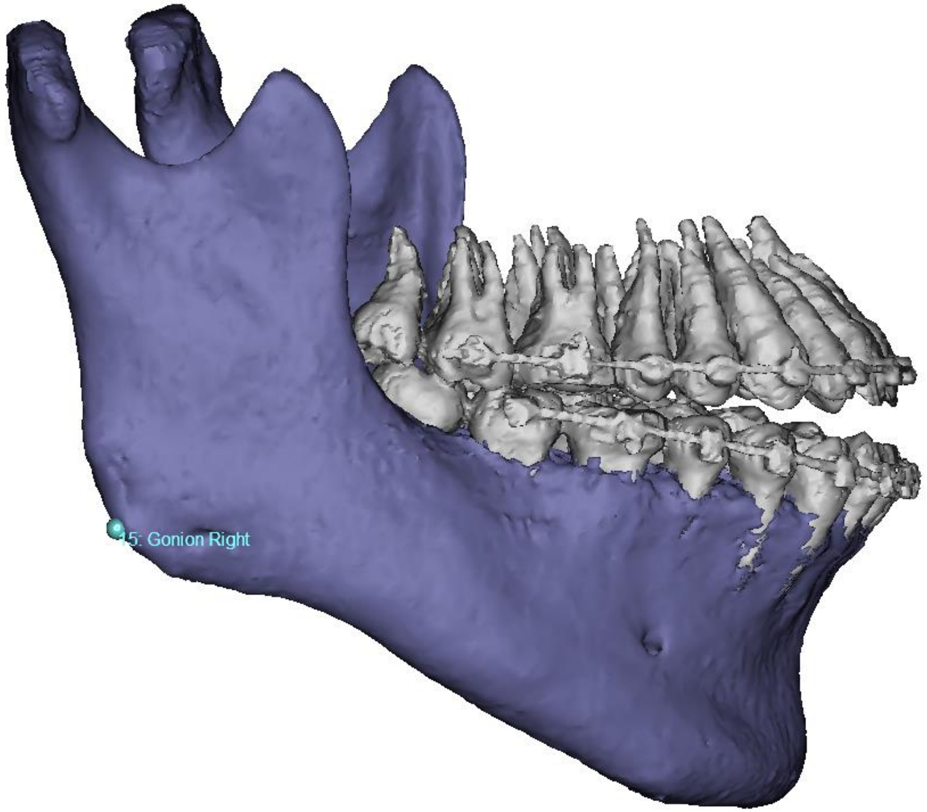

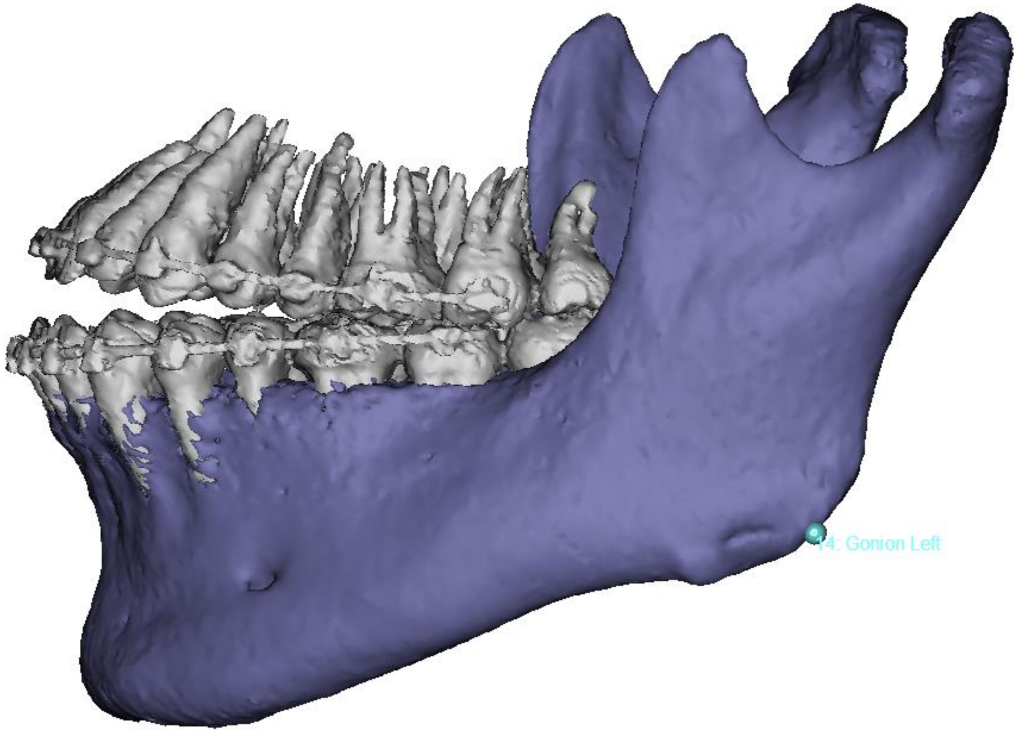

16, 17: Porion L/R	External & uppermost point of the auditory canal L/R	3D view, inferior. At the entry of auditory canal

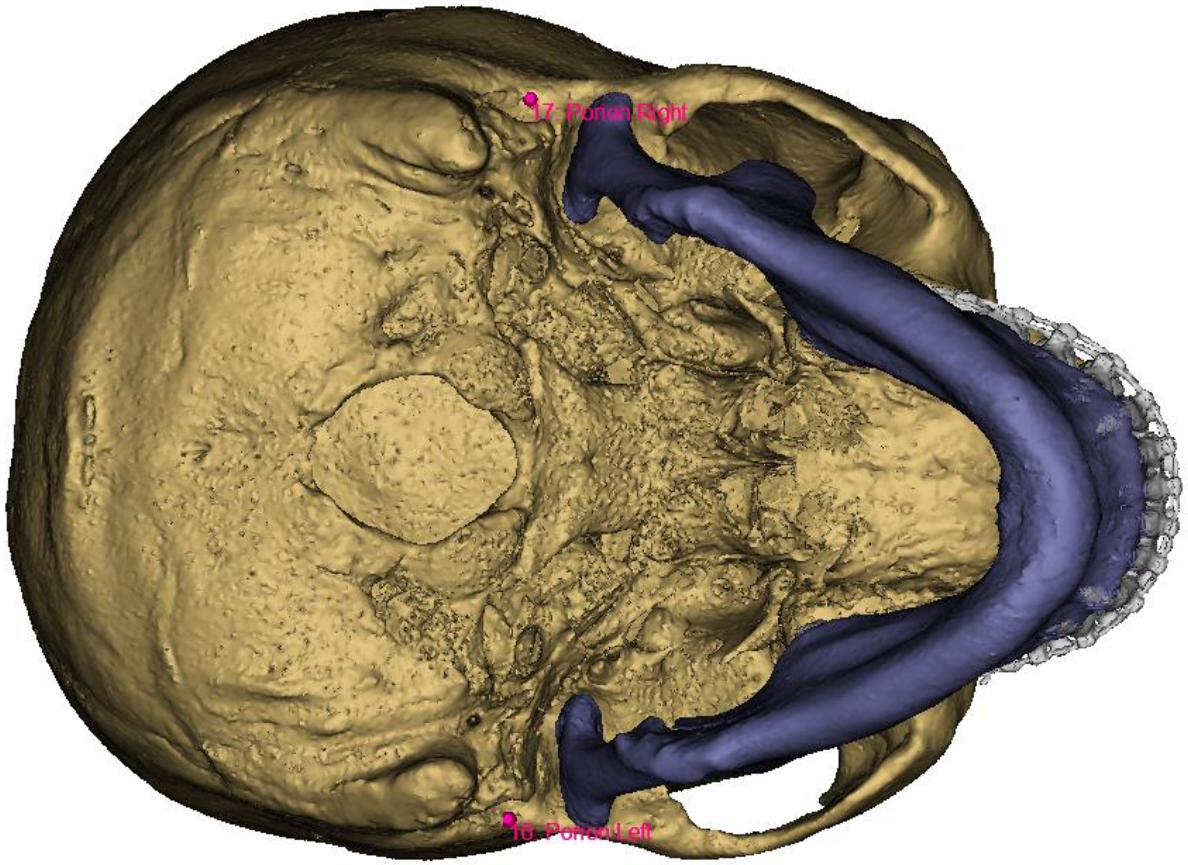

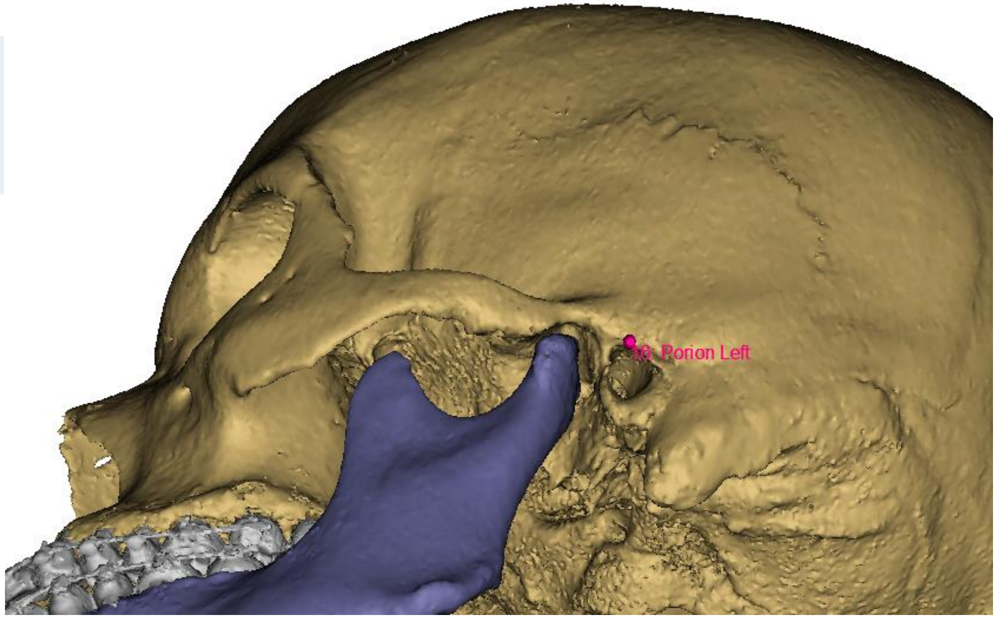

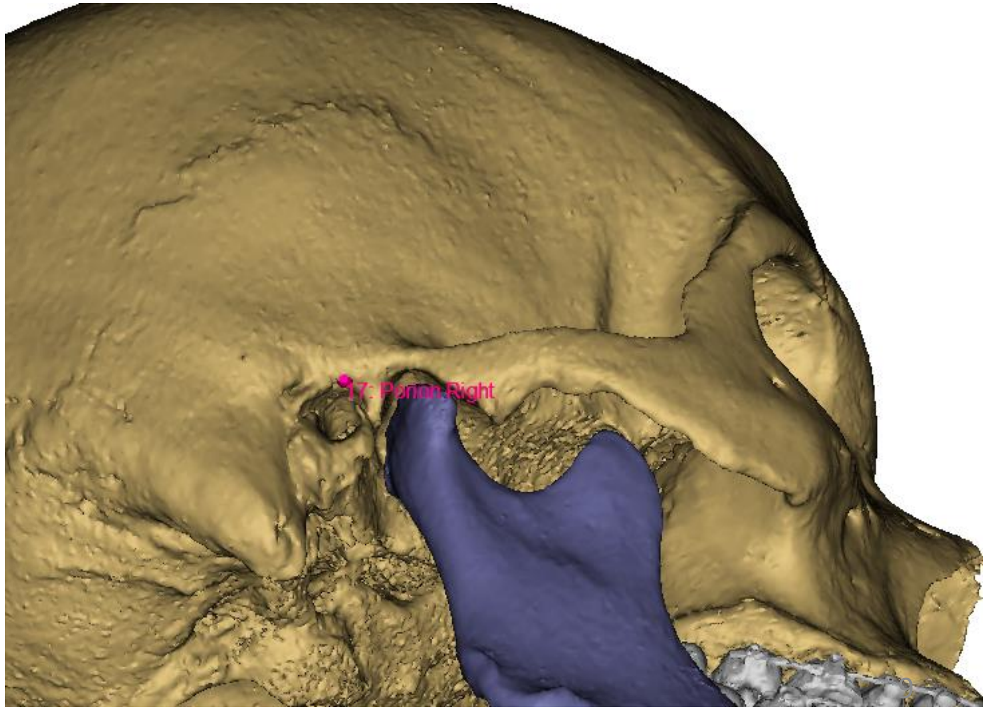

18: PNS	Medial & most distal point of the osseous palate	3D view, inferior-posterior<br>Check on 2D slice (sagittal)

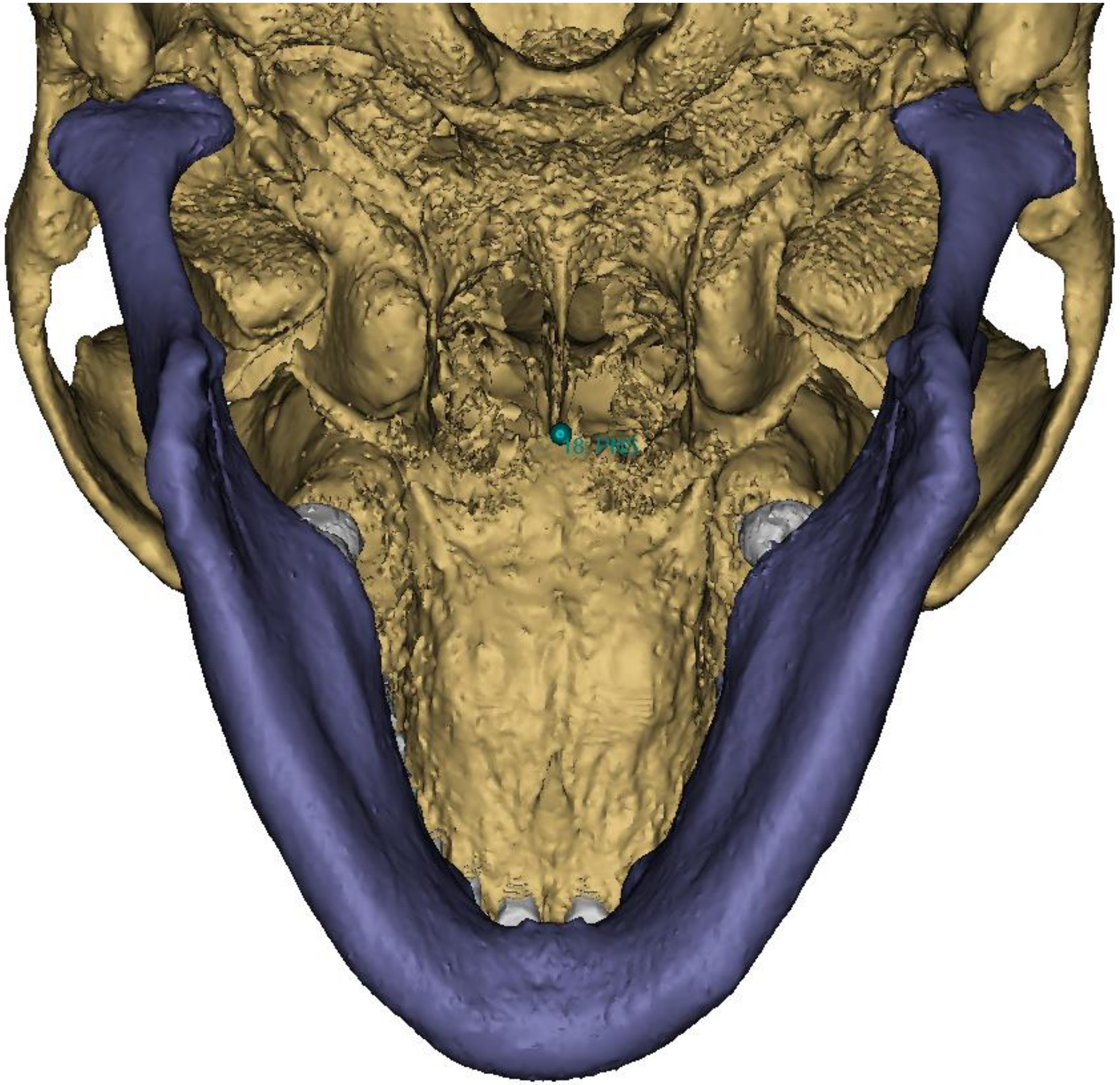

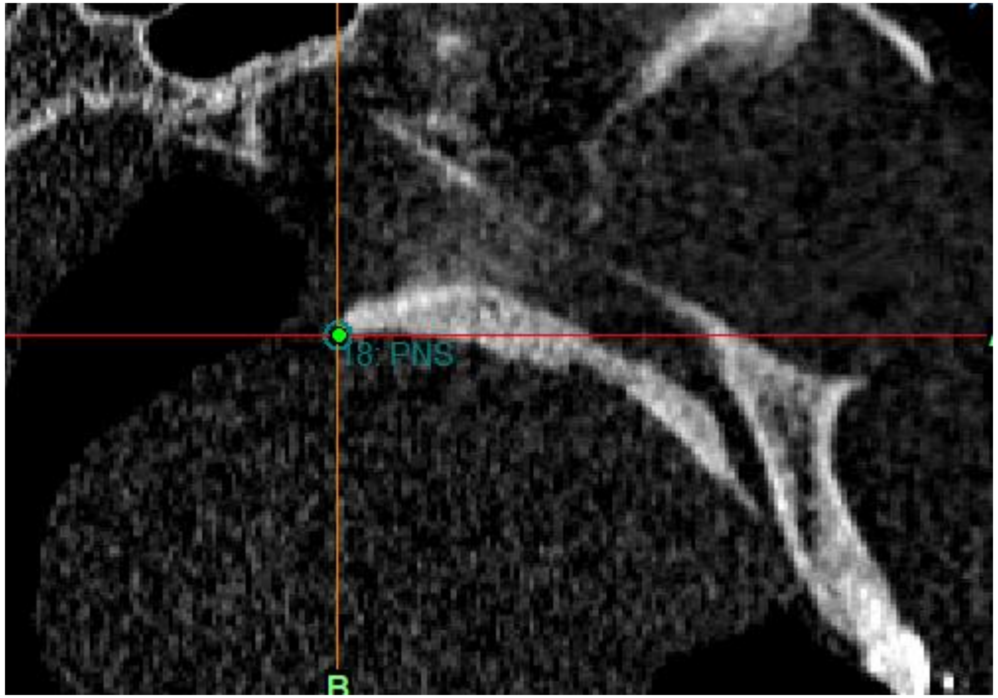

In the right lateral panel, hide the skull and align the 3D lateral view on the Frankfort plane automatically shown in red. **Check the landmarks 7, 8, 9, 10, 11 laterally:**

1/ 3D view, lateral, reorient to have Frankfort plane horizontal

For A point, show the skull

Check position of the points, correct them if necessary.

This can be done on the 2D slices (sagittal)

2/ 3D view, frontal, check that the medial position and alignment of the points is still OK

Afterwards, hide Frankfort plane (glasses icon) and show the skull

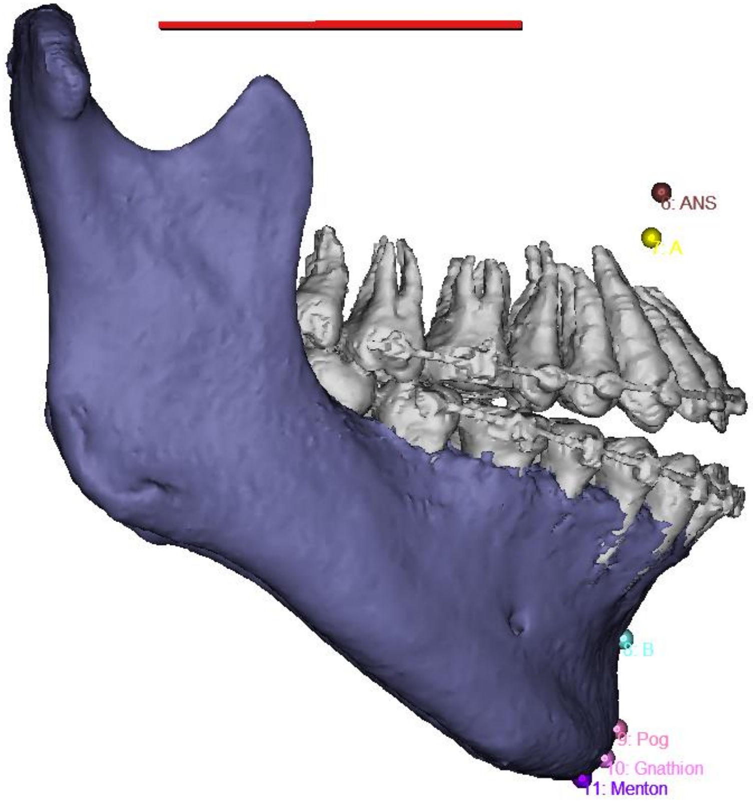

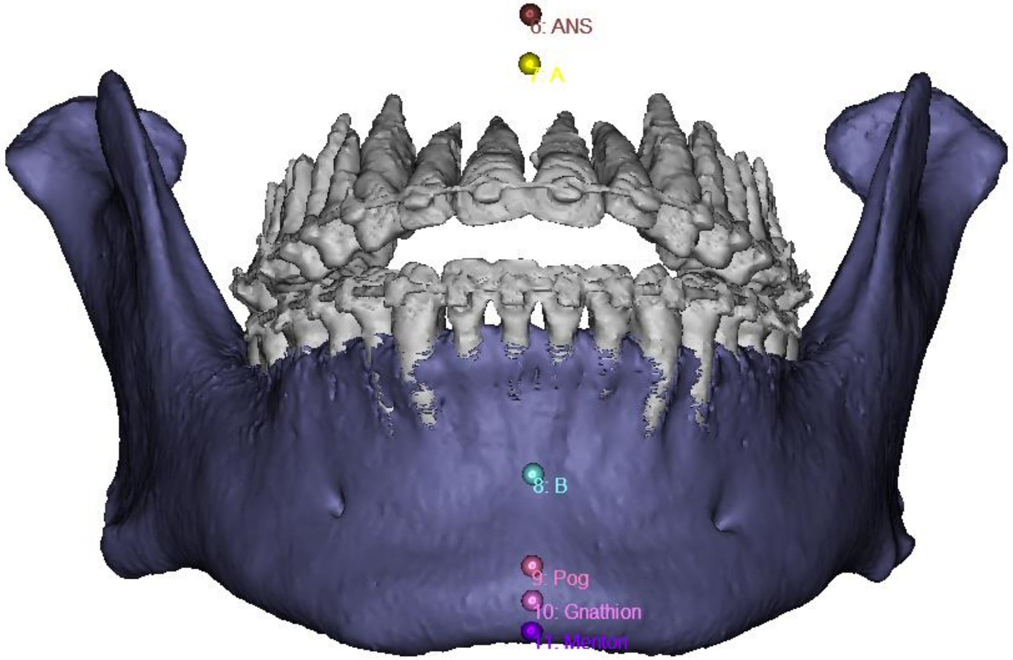

19, 20: Internal Acoustic Foramen L/R	External, most mesial and posterior point of the acoustic foramen L/R	3D view, ¾ postero-mesio-lateral view<br>Check on 2D slice (Axial)

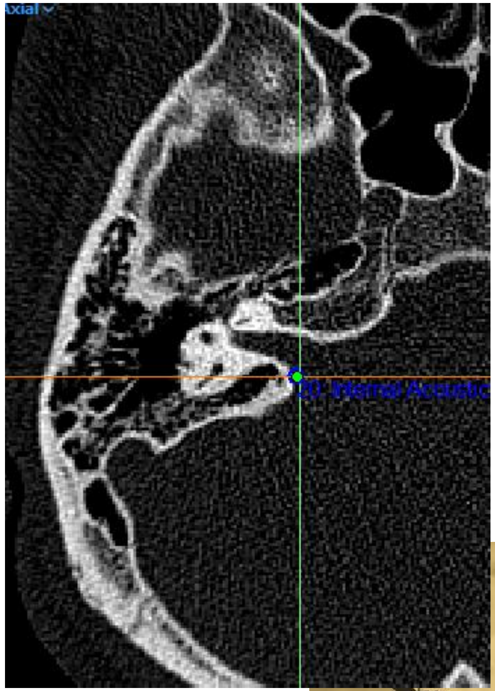

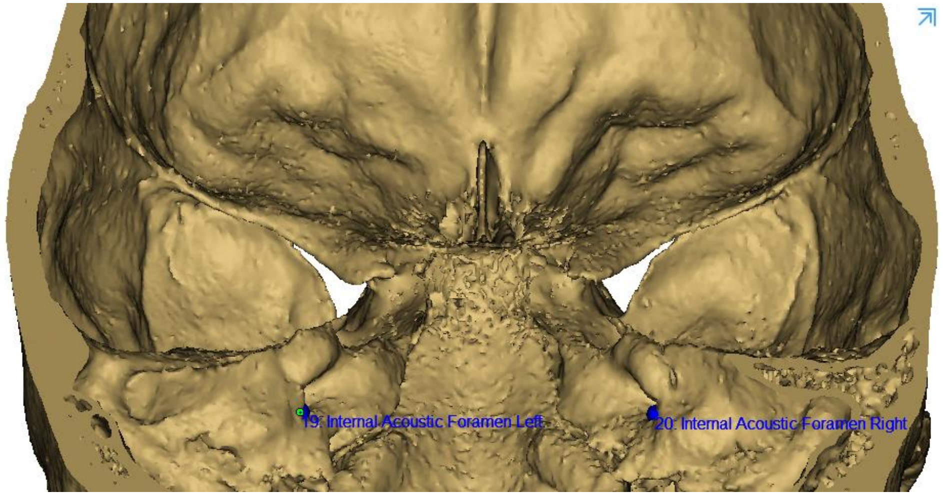

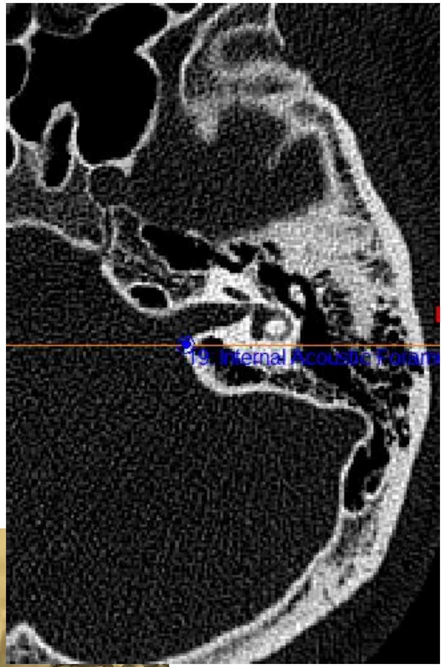

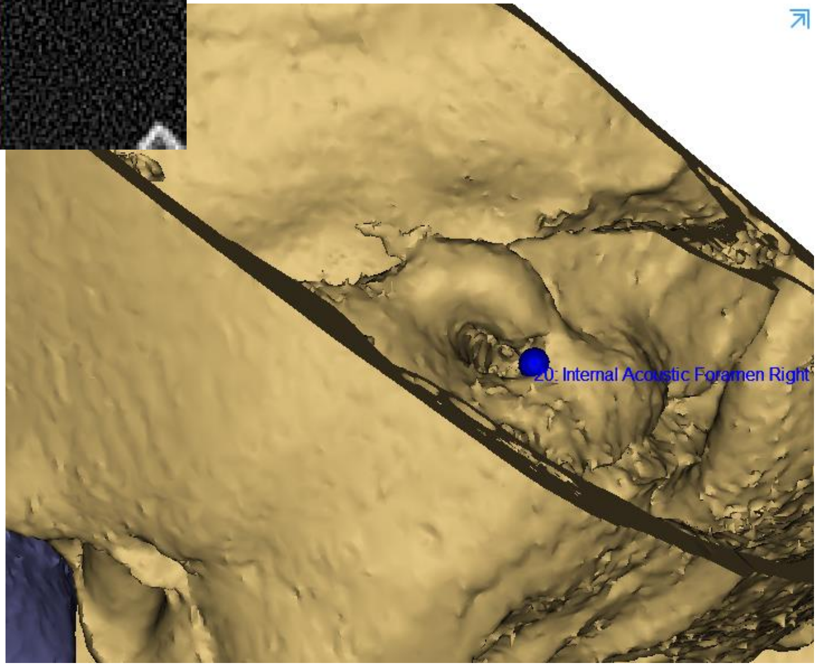

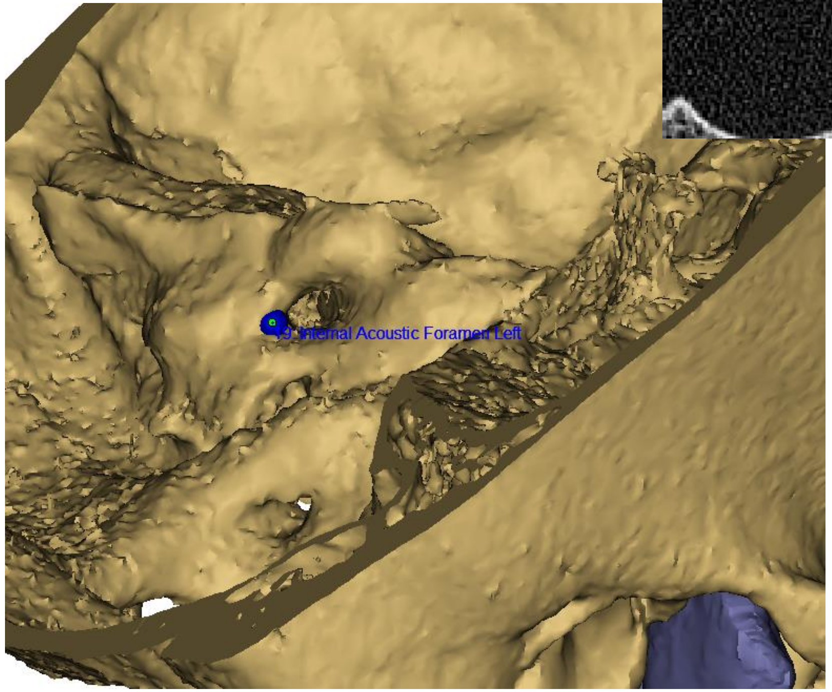

21: Sella	Central point of the sella	2D slices, first sagittal and refine on axial & coronal slices

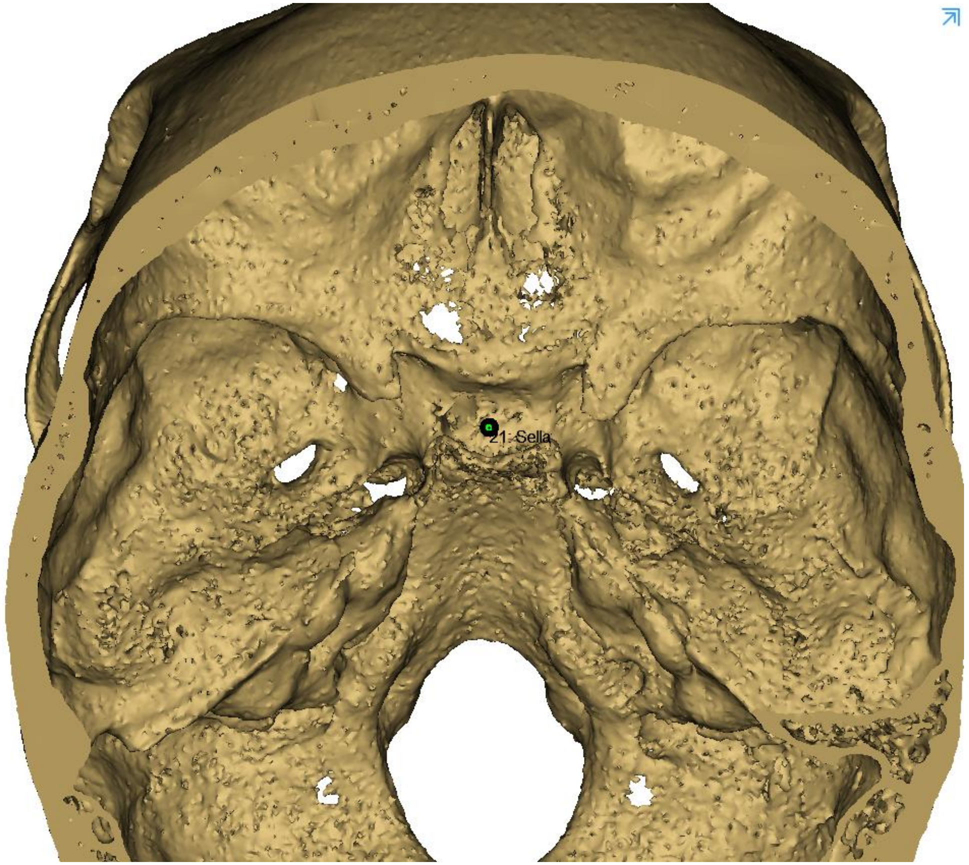

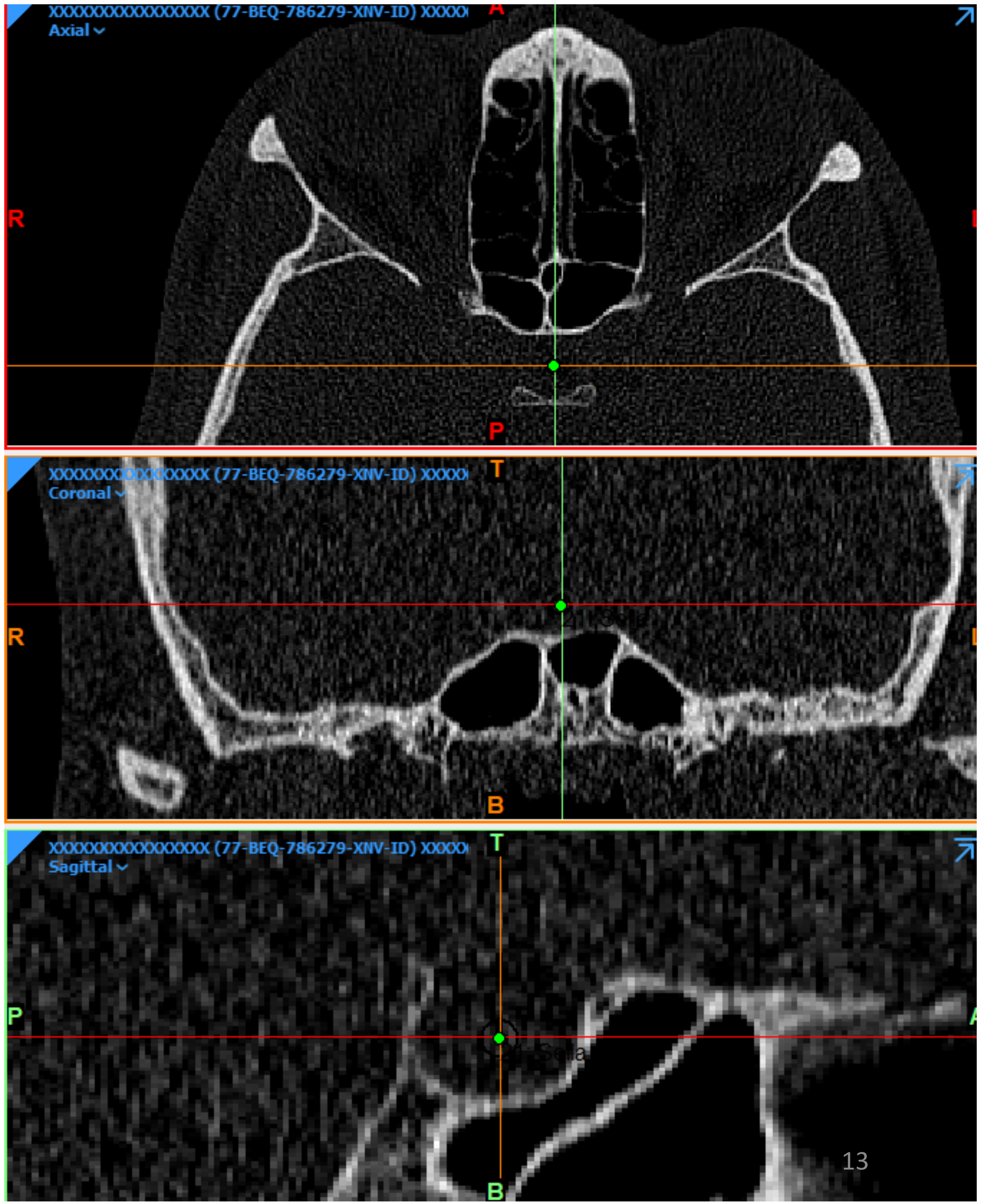

Hide all the landmarks localized (glasses icon)
Hide Upper Skull, Mandible and Lower teeth objects

22, 23: 11E, 21E	Mid-point of 11/21 incisal edges	3D view, inferior
24,25: 16O, 26O	Summit of the mesio-palatal cusp 16/26	3D view, inferior

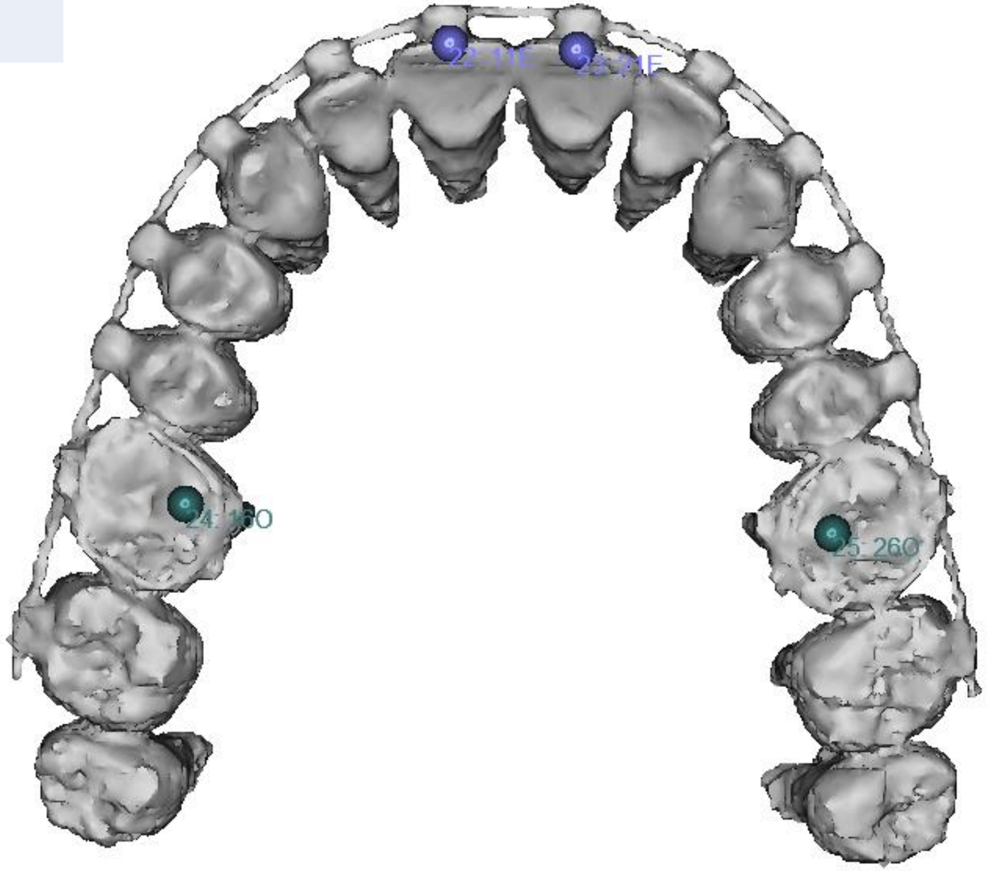

26, 27: 11A, 21A	Root apex of 11/21	3D view, superior

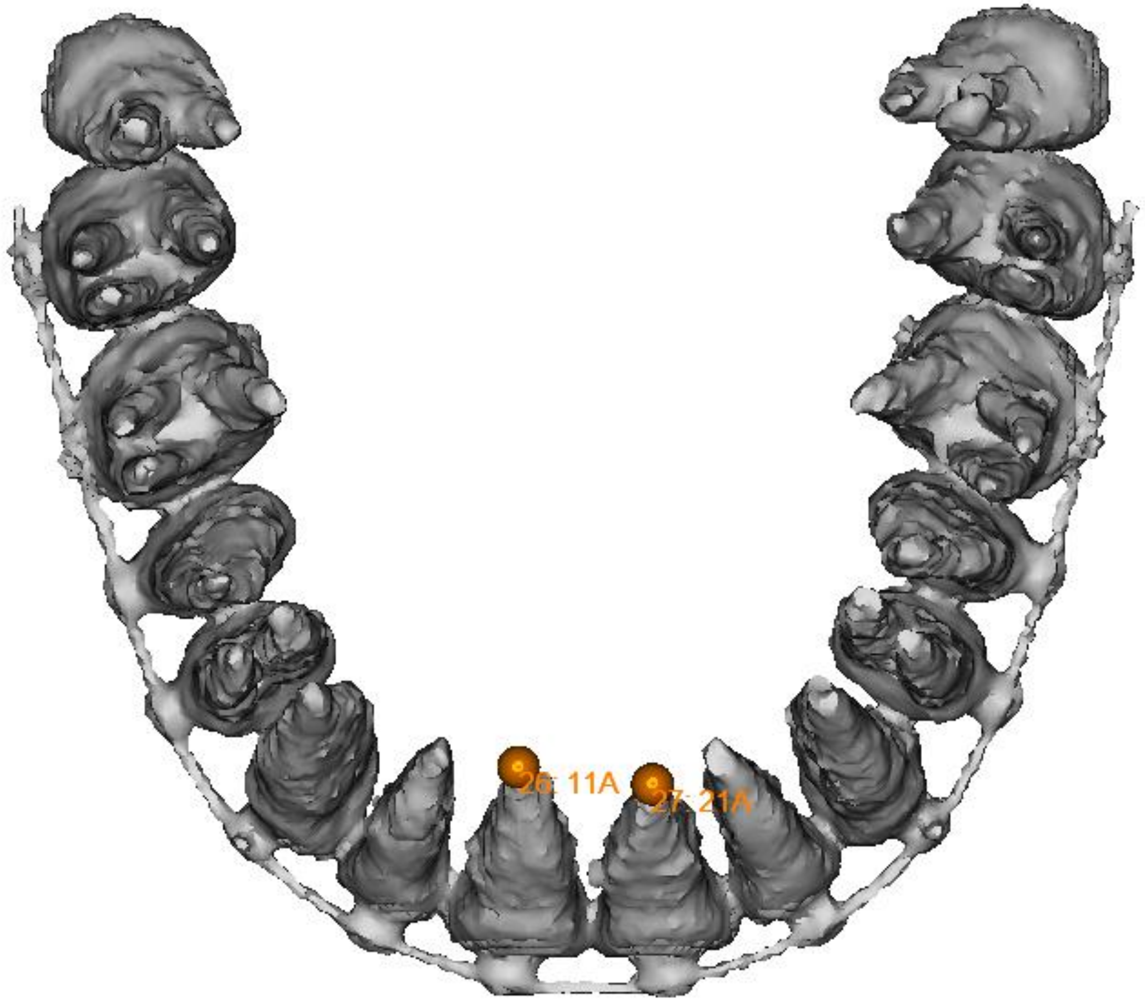

28, 29: 31E, 41E	Mid-point of 31/41 incisal edgess	3D view, superior
30, 31: 360, 460	Central fossa of 36/46	3D view, superior

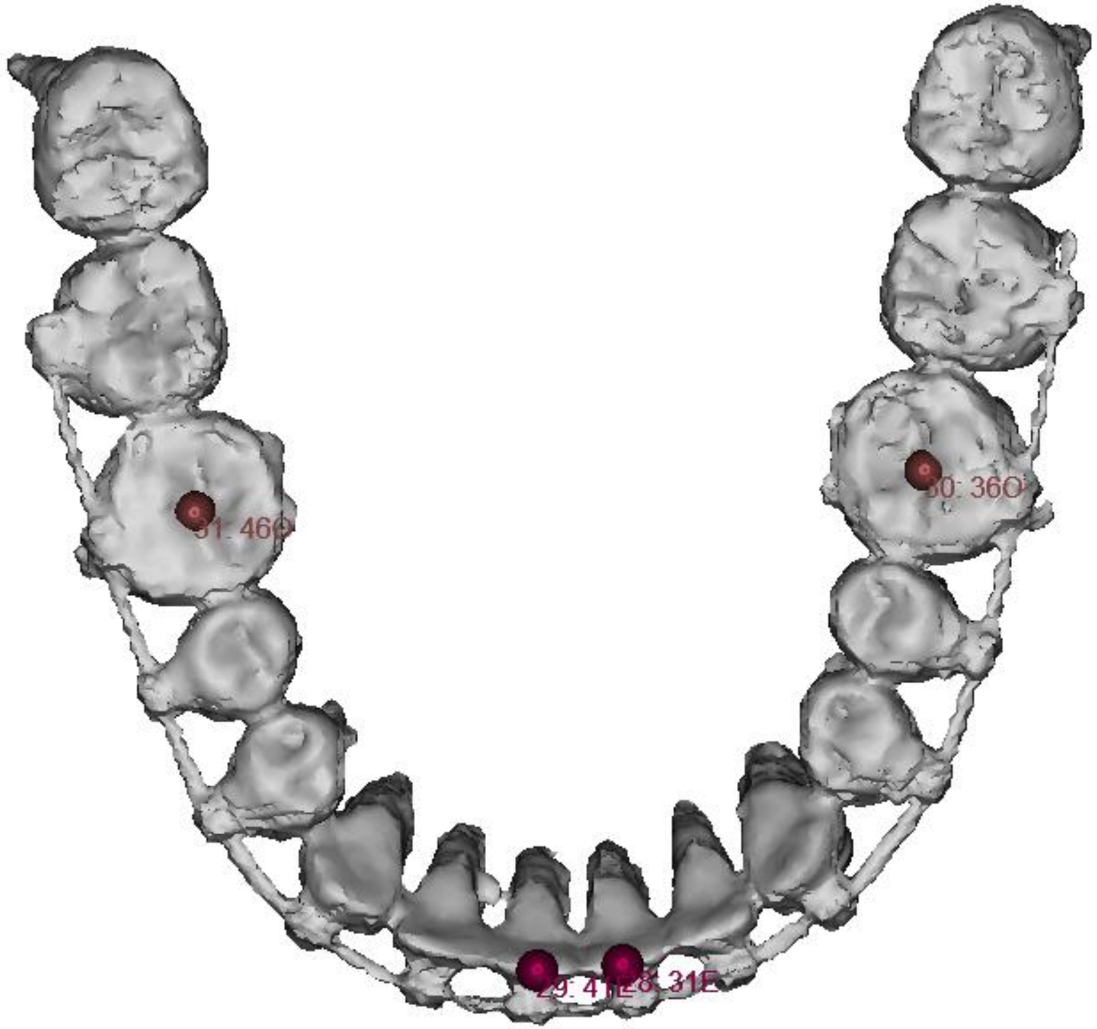

32, 33: 31A, 41A	Root apex of 31/41	3D view, inferior

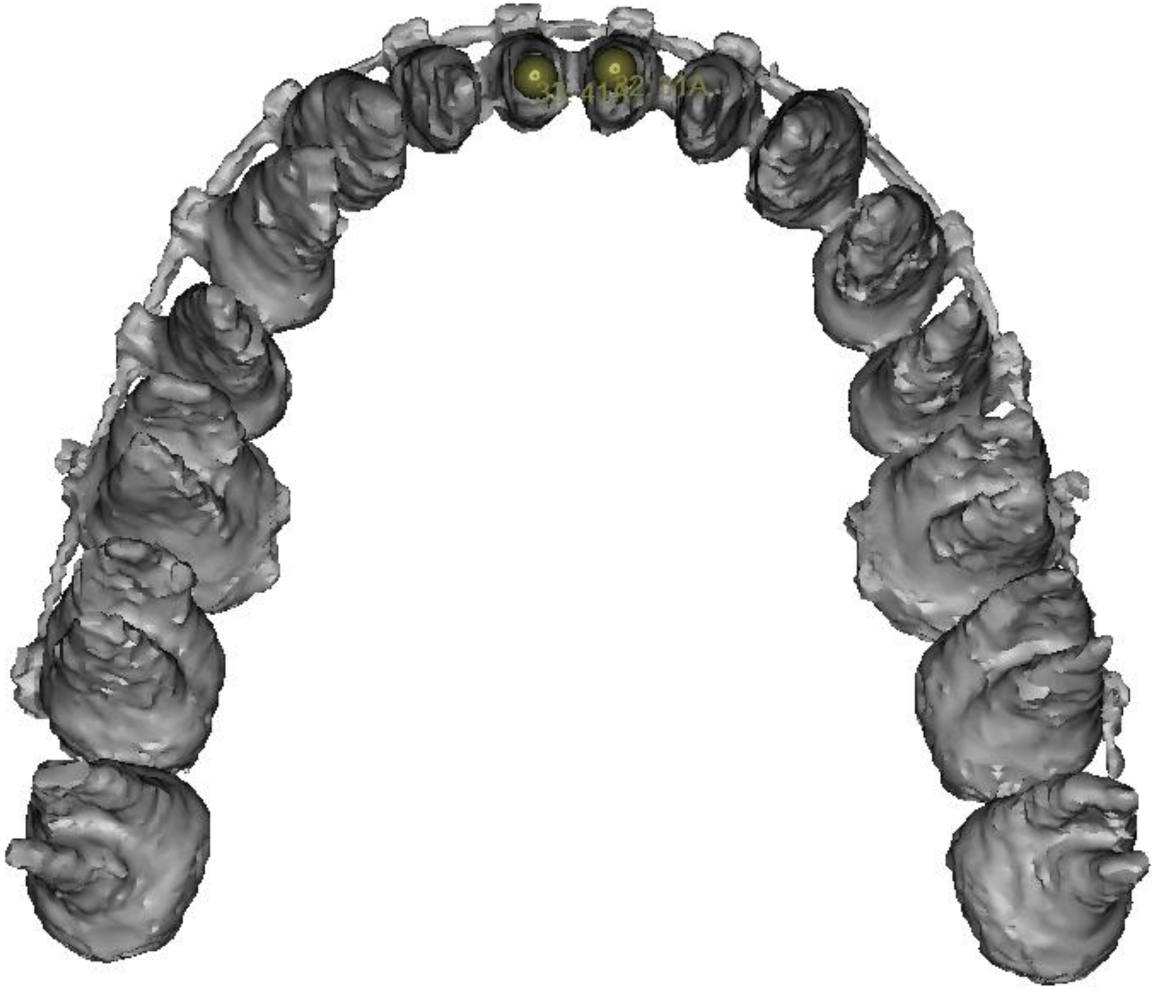

- Stop Time measurement (chronometer)
- Export the xml file using the “export” option of Measure & Analyze window

Thank you ☺

Three-dimensional cephalometric landmarking and Frankfort horizontal plane construction: reproducibility of conventional and novel landmarks

Dot, G.; Rafflenbeul, F.; Kerbrat, A.; Rouch, P.; Gajny, L.; Schouman, T.

J. Clin. Med. 2021

Supplementary Materials

- Supplementary Materials 2: Analysis of Outliers
- Supplementary Materials 3: Angular distances between conventional and novel FH planes

Supplementary Materials 2: Analysis of outliers

Method. For each landmark, repeatability and reproducibility standard deviations (SD) were computed according to the ISO 5725 standard (24) of the International Organization for Standardization. Upon first inspection of the results, the standard's recommendations were followed for clear outlier points, whose annotations were considered as missing data.

Results. We inspected all modified Bland-Altman graphs in order to find possible clear outliers. The only outliers were found for mental foramen points right/left localized by operator #3 during the first annotation session (subjects 4 to 20). Figure SM1 shows Bland-Altmann graphs of the results with these outliers.

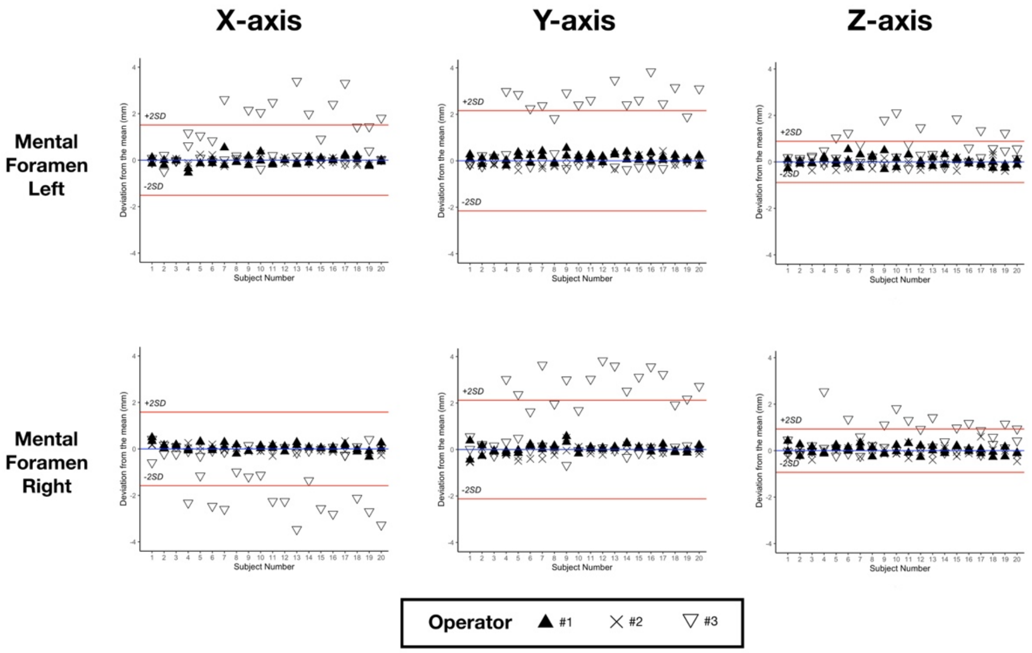

Figure SM1: Bland-Altman plots for mental foramen left and right with outliers, showing the deviations from the mean (blue line) of the 6 repetitions for the 20 subjects. Red lines show the ± 2*SD of reproducibility. SD, standard deviation.

Qualitative analysis of the results showed that the concerned landmarks had been localized in the most distal part of the mental foramina, contrary to the agreed upon definition which demanded placing these landmarks on the most mesial side of the foramen. Consistently with ISO 5725 recommendations, the corresponding annotations were treated as missing data. Figure SM2 shows Bland-Altmann graphs of the results after removal of the outliers.

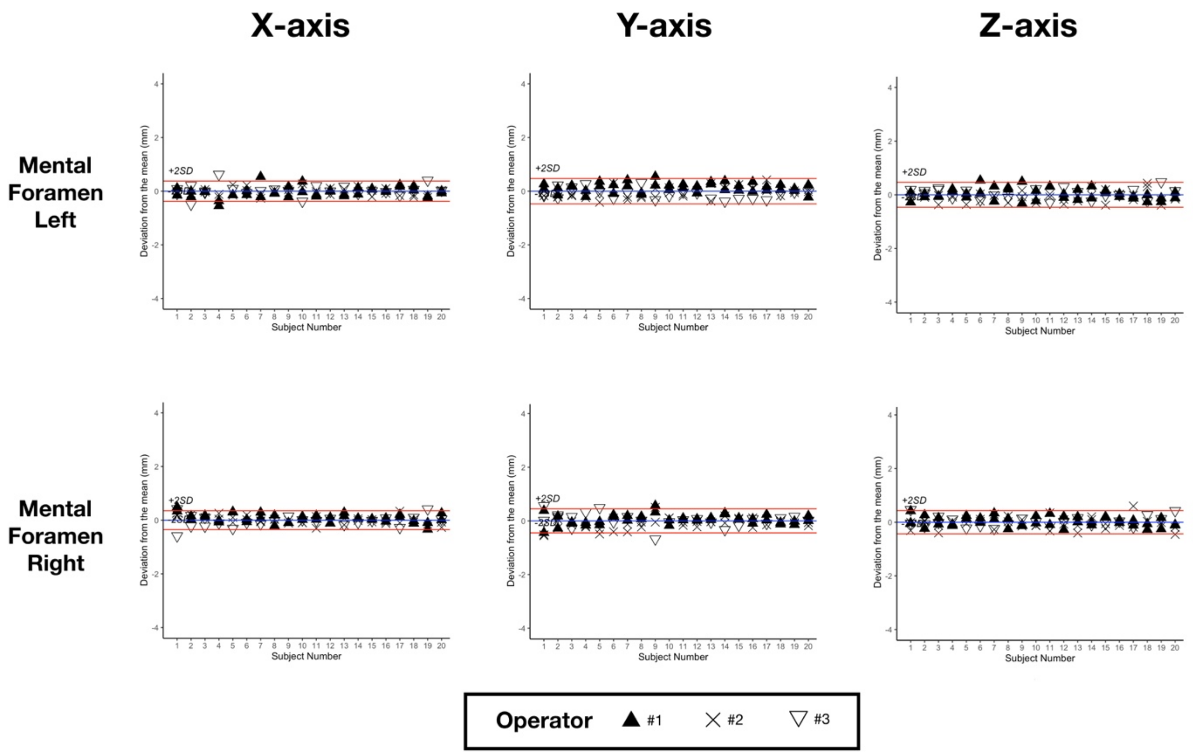

Figure SM2: Bland-Altman plots for mental foramen left and right without outliers, showing the deviations from the mean (blue line) of the 6 repetitions for the 20 subjects. Red lines show the ± 2*SD of reproducibility. SD, standard deviation.

Supplementary Materials 3: Angular distances between conventional and novel FH planes

Method. For each CT scan, four planes were computed using the means of all our operators' observations. Plane definition and labelling followed Pittayapat *et al.*'s publication: FH 1, FH 2, Plane 1 and Plane 3 (Table ST1). The absolute angular differences between each pair of planes were then computed, using trigonometry to calculate the angles between the normals to the planes.

**Plane**	**Definition**
Frankfort horizontal plane 1 (FH 1)	FH by connecting mid-Po, Or-R and Or-L
Frankfort horizontal plane 2 (FH 2)	FH by connecting mid-Or, Po-R and Po-L
Plane 1	A plane connecting mid-Or, IAF-R, IAF-L
Plane 3	A plane connecting Or-R, Or-L and mid-IAF

Table ST1: Definition of the 4 planes used in supplementary analysis

Results. Absolute angular distances between each pair of planes are summarized in Table ST2.

	Angular measurement (°)	
Plane	Mean	SD
FH1 – FH2	0.98	0.57
FH1 – Plane 1	2.94	1.34
FH1 – Plane 3	2.04	1.34
FH2 – Plane 1	2.59	1.27
FH2 – Plane 3	2.41	1.21

Table ST2: Mean absolute angular differences and standard deviations between each pair of planes. FH, Frankfort horizontal plane; SD, standard deviation
